# Progress of potential drugs targeted in lipid metabolism research

**DOI:** 10.3389/fphar.2022.1067652

**Published:** 2022-12-16

**Authors:** Kai Liang, Jian-Ye Dai

**Affiliations:** ^1^ School of Life Science, Peking University, Beijing, China; ^2^ School of Pharmacy, Lanzhou University, Lanzhou, China; ^3^ Collaborative Innovation Center for Northwestern Chinese Medicine, Lanzhou University, Lanzhou, China

**Keywords:** lipid metabolism, fatty acid, small molecule inhibitor, metabolic disease, cancer

## Abstract

Lipids are a class of complex hydrophobic molecules derived from fatty acids that not only form the structural basis of biological membranes but also regulate metabolism and maintain energy balance. The role of lipids in obesity and other metabolic diseases has recently received much attention, making lipid metabolism one of the attractive research areas. Several metabolic diseases are linked to lipid metabolism, including diabetes, obesity, and atherosclerosis. Additionally, lipid metabolism contributes to the rapid growth of cancer cells as abnormal lipid synthesis or uptake enhances the growth of cancer cells. This review introduces the potential drug targets in lipid metabolism and summarizes the important potential drug targets with recent research progress on the corresponding small molecule inhibitor drugs. The significance of this review is to provide a reference for the clinical treatment of metabolic diseases related to lipid metabolism and the treatment of tumors, hoping to deepen the understanding of lipid metabolism and health.

## Introduction

**FIGURE 1:**
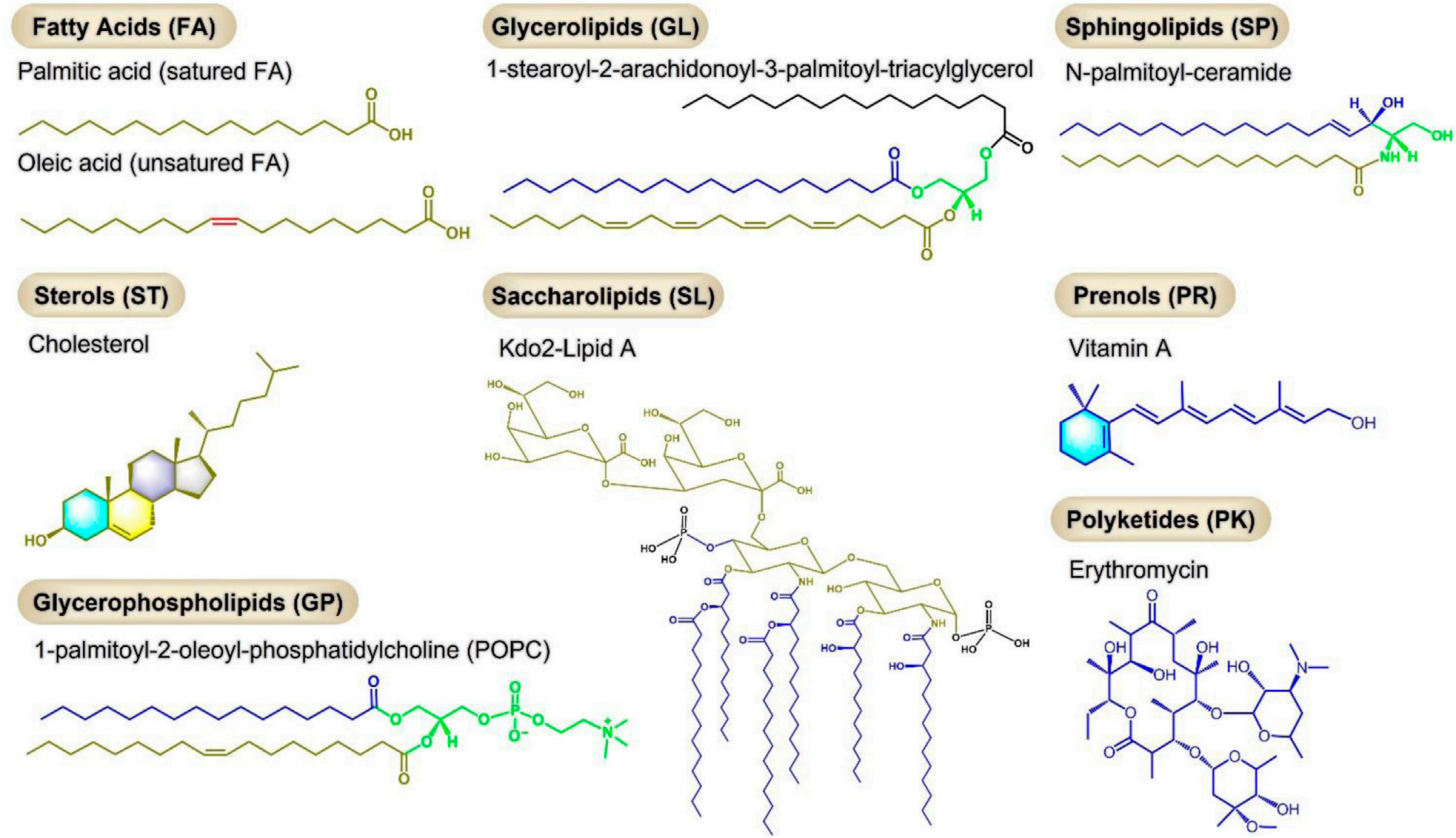
Eight types of lipids, each with a representative molecule.

Lipids are a class of hydrophobic or amphiphilic small molecules which can be divided into eight types: 1) fatty acids, 2) glycerolipids, 3) sphingolipids, 4) sterols, 5) saccharolipids, 6) prenols, 7) glycerophospholipids, 8) polyketides (Figure 1) (Fahy et al., 2007). The diversity of lipids endows them with different biological functions. As one of the three major human nutrients, lipids play an important role in nutrition and health and are closely related to diseases. However, the incidence of abnormal lipid metabolism has gradually increased with the improvement of people’s living standards and changes in dietary habits and lifestyles in recent years. Abnormal lipid metabolism plays an important role in metabolic dysfunction with a variety of diseases, including cardiovascular diseases, diabetes, obesity, non-alcoholic fatty liver disease (NAFLD), non-alcoholic steatohepatitis (NASH), neurodegenerative diseases and cancer (Lim et al., 2014; Butler et al., 2020; Chew et al., 2020).

### Lipid metabolism with cardiovascular diseases

Abnormal lipid metabolism is a big risk factor for cardiovascular diseases (McGranaghan et al., 2021). Lipid metabolism is closely related to the occurrence and development of atherosclerosis. Atherosclerosis is a common clinical disease closely related to coronary heart disease and cerebral infarction (Pothineni et al., 2017). Although there have been related therapeutic drugs (such as statins), their clinical use is limited by their limited efficacy and side effects. Therefore, the search for novel therapeutic drugs of the cardiovascular diseases remains urgent.

### Lipid metabolism with diabetes

Worldwide, about 537 million people suffer diabetes, and type 2 diabetes (T2D) accounts for 90% of diabetes patients, which has become a serious health risk (Ahmad et al., 2022). Obesity is a major risk factor for T2D (Kahn et al., 2006). In obese patients, elevated triglyceride levels lead to increased levels of free fatty acids, which can lead to insulin resistance and glucose intolerance (Boden, 2003). The body metabolizes glucose inefficiently and lipolysis increases, releasing free fatty acids and glycerol, accompanied by an increase in fatty acid β-oxidation (FAO). This leads to the accumulation of large amounts of ketones and the production of large amounts of ketone bodies such as acetoacetic acid, beta-hydroxybutyric acid, and acetone, resulting in diabetic ketoacidosis (Dhatariya et al., 2020). In addition, FAO (especially very long chain fatty acids) may mediate the increase in diabetes-induced oxidative stress, which leads to the development of diabetic complications (Giacco and Brownlee, 2010). Conventional hypoglycemic drugs predispose patients to a wide range of side effects, such as cardiovascular risk and weight gain. In mice with pharmacological inhibition or adipose-specific deletion of (adipose triglyceride lipase) ATGL, hormone-sensitive lipase (HSL), or monoacylglycerol lipase (MAGL), free fatty acids from adipose tissue lipolysis was reduced, resulting in a significant increase in glucose tolerance and improved insulin sensitivity (Roden and Shulman, 2019). Intervening lipid metabolism may be a potential approach to treating diabetes.

### Lipid metabolism with NAFLD and NASH

NAFLD is a common chronic liver disease, affecting at least 1 in 4 adults worldwide (Pappachan et al., 2017). NASH is the progressive stage of NAFLD, which can progress to cirrhosis and even hepatocellular carcinoma (Wree et al., 2013). There are currently no approved drugs to treat NASH. Potential drugs to correct abnormal lipid metabolism related to NAFLD and NASH include acetyl-CoA carboxylase (ACC) inhibitors, stearoyl-CoA desaturase-1 (SCD1) inhibitors, fatty acid synthase (FASN) inhibitors, and so on.

### Lipid metabolism with cancer

Lipid metabolism dysregulation is one of the most prominent metabolic changes in cancer. Enhanced lipid synthesis or uptake contributes to the rapid growth of cancer cell and tumor formation (Munir et al., 2019). Cancer cells use lipid metabolism to obtain energy and membrane components needed for proliferation and metastasis. FAO is the prefered energy source of cancer cell after the presence of drug-resistance (Oren et al., 2021), and restricting this process can inhibit cancer development. In recent years, the research of small molecule drugs targeting lipid metabolism pathway has become the trend of cancer therapy.

### Lipid with neurodegenerative diseases

Much more studies have shown that lipid metabolism is involved in the occurrence and development of a variety of neurodegenerative diseases, especially in the pathogenesis of Alzheimer’s disease (AD) and Parkinson’s disease (PD) (Nury et al., 2020). But the mechanism that abnormal lipid metabolism leads to neurodegenerative diseases has long been a mystery. Targeting FASN, Diacylglycerol O-acyltransferase 1 (DGAT), ATP-citrate lyase (ACLY) and maybe other important proteins appears to alleviate neurodegenerative diseases to some extent. Further research is urgently needed.

Nowadays, lipid research with health has become a research hotspot at home and abroad. This review summarizes important key proteins in the lipid metabolism process that can be developed into drug targets, such as carnitine palmitoyl-transferase 1 (CPT1), ACLY, FASN, and presents recent progress in the development of potential small molecule drugs. These targets are involved in processes such as lipid uptake, synthesis, oxidation and are closely related to metabolic diseases. Given the important physiological role of lipid metabolism, a growing number of scientists and pharmaceutical companies are focusing on the development of drugs that target lipid metabolism.

## Drug targets in lipid uptake

### Fatty acid uptake

Fatty acids are the simplest lipids and are essential components of complex lipids. Mammals produce only a limited number of fatty acids. Other fatty acids, especially polyunsaturated, must be obtained from the diet (Dyall et al., 2022). Fatty acids come from two sources: extracellular uptake through specific proteins on the cell membrane and lipolysis of intracellular lipid droplets (Grabner et al., 2021). Extracellular fatty acids (long chain) uptake entering into cytosol must be aided by several membrane proteins, like CD36 (Cluster of Differentiation 36, also named fatty acid translocase), and long-chain fatty acid transport proteins (FATP) (Ma et al., 2021).

### CD36 and FATP

CD36 is overexpressed in various cancer cells and is critical for cancer cell metastasis (Wang and Li, 2019). Blocking of CD36 almost stops the migration of oral cancer cells in mouse models, and some other cancer cell metastasis can also be impaired (Pascual et al., 2017). A recent study implies that CD36-mediated free fatty acid uptake is essential for hematopoietic stem cells (HSC) in response to acute infection, which can switch HSC metabolism from anaerobic glycolysis to fatty acid β-oxidation, thereby satisfying energy demands from HSC expansion and differentiation (Mistry et al., 2021). A study by the Memorial Sloan Kettering Cancer Center shows that treatment with an Inhibitor targeting FATP can block lipid transport into melanoma cells, thereby reducing melanoma cell growth and infection (Zhang et al., 2018). Descriptions of CD36 and FATP-related small molecule inhibitors are summarized in Table1.

**TABLE 1:** Potential pharmacological targets and inhibitors targeting lipid uptake.

Drug target	Notable inhibitors	Inhibitor description	IC50	Development status	Related diseases	Chemical structure	References
FATP2	Lipofermata	—	4.84 μM	Preclinical Stage	Melanoma	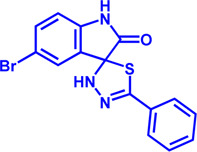	Zhang et al. (2018)
CD36	ABT-510	TSP-1 mimetic drug	—	Phase 2	Melanoma; Renal cell carcinoma, Lymphoma; Glioblastoma; Brain Tumor	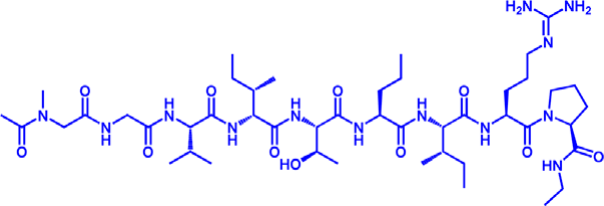	Campbell et al. (2010), Markovic et al. (2007), Nabors et al. (2010)
MAGL	ABX-1431	An first-in-class irreversible inhibitor	8 nM	Phase 2	Neurological disorders	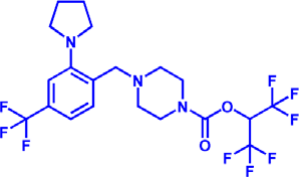	Cisar et al. (2018)
MAGL	MJN110	Irreversible	9.1 nM	Preclinical Stage	Diabetes; Neuropathy	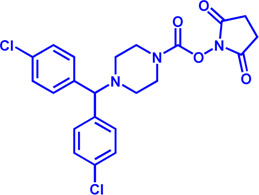	Wilkerson et al. (2016)
MAGL	JNJ-42226314	Highly selective; Non-covalent; Reversible	1.13 nM (Hela cells)	Preclinical Stage	Neuropathic and inflammatory pain	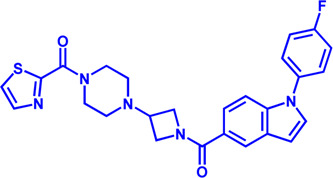	Wyatt et al. (2020)
MAGL	JZL184	The first selective MAGL inhibitor	8 nM	Preclinical Stage	T2D; Glioblastoma	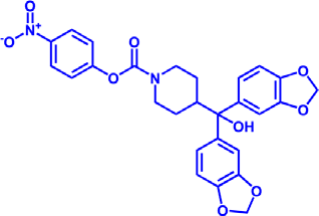	Taib et al. (2019), Walenna et al. (2020)
NPC1L1	Ezetimibe	a selective inhibitor; Oral	—	FDA approved	Primary hyperlipidemia; Familial cholesterolemia	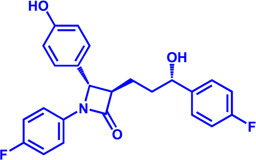	Long et al. (2021), Rocha et al. (2022)

The chemical information of small molecules is collected from Pubchem database (https://pubchem.ncbi.nlm.nih.gov), and 2D structures in the above diagram were drawn by Chemdraw software. IC50, half maximal inhibitory concentration.

Another important source of fatty acids is lipid droplets. Lipid droplets not only store energy but also participate in catabolism for energy as needed. Lipolysis is a well-known metabolic process that releases fatty acids through three sequential catalysis by ATGL (the rate-limiting step of the triglyceride lipolysis process), HSL, MAGL (Zimmermann et al., 2004).

### MAGL

Inhibition of MAGL significantly reduces the occurrence of inflammation and neurodegeneration (Deng and Li, 2020). In addition, the overexpression of MAGL is present in various cancers such as breast cancer and is closely related to the proliferation of cancer cells (Deng and Li, 2020). Therefore, the development of small molecule inhibitors targeting MAGL could serve as potential drugs for the treatment of neurological disorders and cancer (Gil-Ordonez et al., 2018). Although many MAGL inhibitors have been developed, there is still a lack of inhibitors with few side effects and high selectivity (Table 1).

### Cholesterol uptake

Cholesterol, a major component of cell membranes, is involved in the synthesis of steroid hormones and also has a variety of physiological roles (Huff et al., 2022). However, excessive cholesterol intake has instead become a direct factor in the increased incidence of hypercholesterolemia and an important cause of diseases such as atherosclerosis and stroke (Reina et al., 2015). Proper regulation of cholesterol levels is essential for human health. Cholesterol uptake includes Niemann–Pick C1-like 1 (NPC1L1) -mediated small intestine cholesterol absorption and low-density lipoprotein receptor (LDLR)-mediated low-density lipoprotein—cholesterol (LDL-C) uptake (Luo et al., 2020). There are three classes of the most important cholesterol-lowering drugs available, namely statins (endogenous cholesterol synthesis inhibitors, described later), NPC1L1 inhibitors and proprotein convertase subtilisin/kexin type 9 (PCSK9) inhibitors (Luo et al., 2020). The three classes of drugs act on different parts of cholesterol metabolism to exert a cholesterol-lowering effect.

### NPC1L1

NPC1L1 plays a central role in intestinal cholesterol absorption and can significantly affect the amount of cholesterol absorbed by the intestine (Zhang et al., 2022). The only NPC1L1 inhibitor currently on the market is ezetimibe (Zhang et al., 2022). Ezetimibe reduces plasma cholesterol levels by inhibiting NPC1L1 activity and decreasing cholesterol absorption in the intestine. As cholesterol levels increase, the sterol-sensing domain (SSD) of NPC1L1 can bind more cholesterol, which in turn induces the formation of SSD structural clusters. The binding of ezetimibe deforms the SSD and disrupts the structural clusters, thus inhibiting NPC1L1 function and lowering cholesterol (Hu M. et al., 2021).

### LDLR-mediated endocytosis

LDL-C enters the cell through LDLR-mediated endocytosis. PCSK9 binds to LDLR and promotes its degradation, reducing the ability of hepatocytes to uptake cholesterol (Luo et al., 2020). PCSK-9 inhibitors significantly inhibit PCSK-9 activity and indirectly lower blood cholesterol levels. Cholesterol-lowering drugs targeting PCSK9 have two mechanisms: blocking the binding of PCSK9 to LDL-R, such as monoclonal antibodies, and inhibiting the expression of PCSK9 molecules or interfering with PCSK9 secretion, such as interfering RNA, antisense oligonucleotides (ASO), and small molecule cyclic peptide inhibitors (Rifai and Ballantyne, 2021).

Currently, there are three drugs marketed worldwide that target the PCSK9 target, of which two are marketed as evolocumab monoclonal antibody and alirocumab monoclonal antibody, which target the binding of PCSK9 to LDL-R (Rifai and Ballantyne, 2021). The other is Inclisiran, a long-acting therapeutic agent developed by Novartis to inhibit PCSK9 expression by means of RNA interference (RNAi) (Samuel et al., 2022). These three PCSK9 inhibitors have the advantages of high specificity and clear mechanism of action, providing a new therapeutic option for cholesterol lowering.

Since most PCSK9 inhibitors that have been marketed and are in clinical development are subcutaneous injections, they are inconvenient to use. As a result, the development of novel oral PCSK9 inhibitors is quite needed. There are several oral PCSK9 inhibitors in the clinical stage, including AZD8233, MK-0616 and NNC0385-0434. AZD8233 is an antisense oligonucleotide that is used for inhibition of PCSK9 mRNA translation and protein synthesis in hepatocytes (Gennemark et al., 2021). Studies have shown that a single injection of AZD8233 can reduce PCSK9 by more than 90% and LDL-C by 70% in people with high cholesterol, and the feasibility of oral administration of AZD8233 has been demonstrated (Gennemark et al., 2021). MK-0616 is a 10 amino acid cyclic peptide PCSK9 inhibitor developed by Merck Sharp & Dohme (Tucker et al., 2021). Result of Clinical Phase 1 showed that taking MK-0616 reduced blood levels of free PCSK9 protein by more than 90%, and cholesterol levels were reduced by approximately 65 percent when combined with a statin for 14 days. This drug is now in Phase II clinical study (ClinicalTrials.gov NCT05261126). NNC0385-0434 is a small molecule peptide PCSK9 inhibitor developed by Novo Nordisk that has a similar structure to LDLR and inhibits PCSK9 binding to LDLR. It is currently in Phase 2 clinical trials (ClinicalTrials.gov Identifier: NCT04992065). In addition, two oral PCSK9 inhibitors from China, CVI-LM001 (clinical phase 2, ClinicalTrials.gov Identifier: NCT04438096) and DC371739 (clinical phase 1, ClinicalTrials.gov Identifier: NCT04927221), have entered clinical studies. CVI-LM001 lowers cholesterol indirectly by reducing the expression of the PCSK9. Preliminary clinical data show that CVI-LM001 reduces the expression level of the PCSK9 gene by 90% and exhibits good pharmacokinetics (Xu et al., 2019). DC371739 impedes pcsk9 expression by binding HNF-1α. Combination with the statin atorvastatin may be a therapeutic strategy for statin-intolerant patients (Wang J. et al., 2022).

Despite the effectiveness of oral PCSK9 inhibitors in lowering cholesterol, their relative low bioavailability requires high doses and daily dosing, resulting in high costs for patients. Future optimization is still needed to better serve patients.

## Drug targets in lipid synthesis

Mammalian lipid synthesis occurs mainly in liver and adipose tissue. Acetyl-CoA from glycolysis and FAO enters the TCA cycle to generate citric acid, which is shuttled into the cytoplasm by mitochondrial citrate carrier solute carrier family 25 member 1 (SLC25A1) and regenerate into acetyl-CoA by ACLY. Acetyl-CoA is then carboxylated to form malonyl-CoA under the catalysis of ACC (the rate-limiting step in the fatty acid synthesis) (Ito et al., 2021). Malonyl-CoA participates in a series of reactions that extend the FA chain by two carbons at a time. Seven malonyl-CoA and one acetyl-CoA are catalyzed to form palmitic acid, followed by chain extension and desaturation.

**FIGURE 2:**
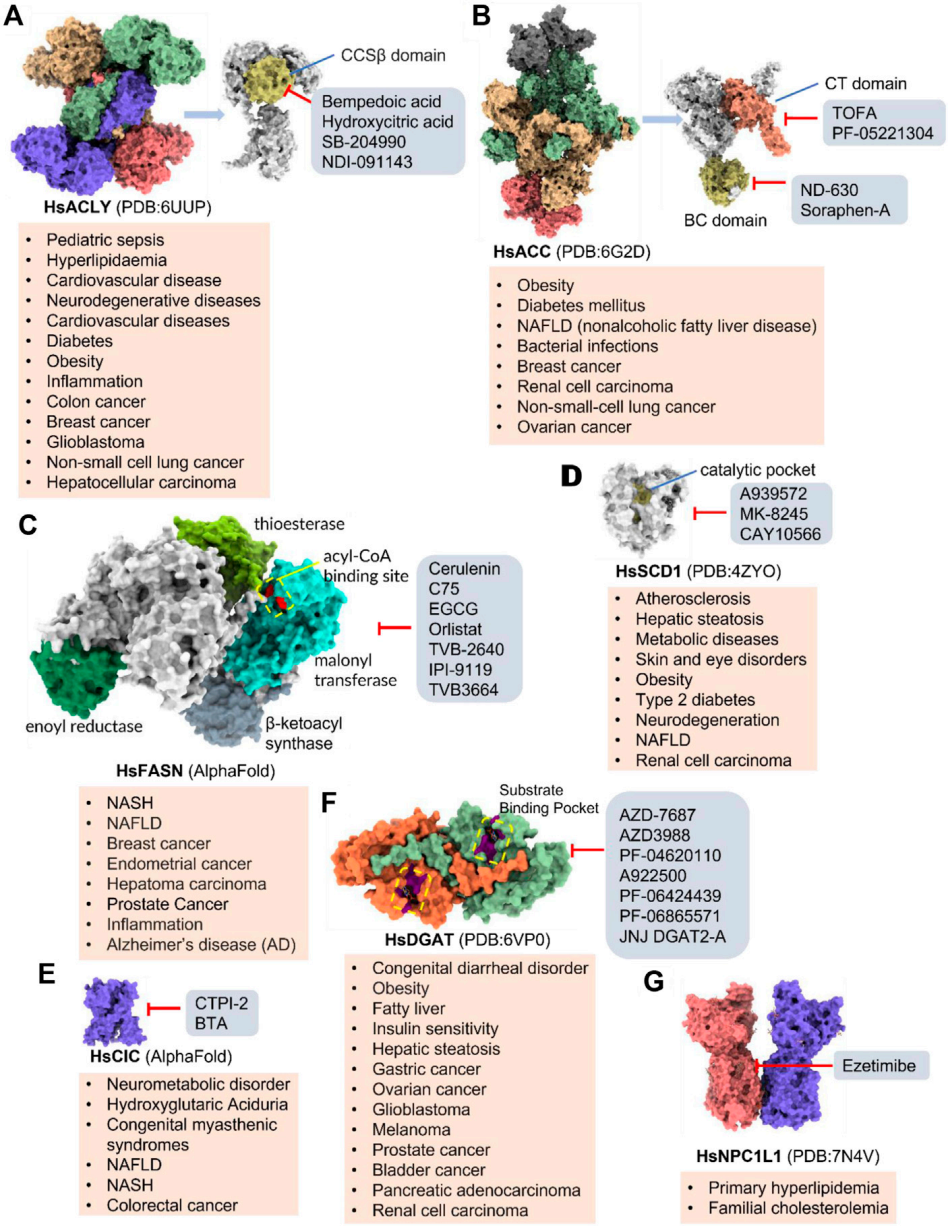
Potential pharmacological targets and related inhibitors targeting fatty acid synthesis and storage, with related diseases are listed under the respective structure model. **(A)** Homotetramer and single subunit structure of human ACLY. Citryl-CoA synthetase (CCS) module highlighted by dark khaki color is the region targeted by most currently known ACLY inhibitors (Bempedoic acid, Hydroxycitric acid, SB-204990, NDI-091143). **(B)** Human ACC filament structure and single subunit structure with two inhibitor targeting regions (BC domain and CT domain) highlighted. Inhibitors ND-630 and Soraphen-A target the BC domain; TOFA and PF-05221304 targets CT domain. **(C)** Predicted structure model of human FASN (from AlphaFold database) with substrate binding site labled. Well-known small molecule inhibitors of FASN (Cerulenin, C75, EGCG, Orlistat, TVB-2640, IPI-9119, TVB3664) are listed on the right. **(D)** Human SCD1 structure, with 3 inhibitor small molecules (A939572, MK-8245, CAY10566) targeting the catalytic pocket. **(E)** Predicted structure model of human SLC25A1 (CIC) from AlphaFold database with its two famous inhibitors (CTPI-2, BTA). **(F)** Structure of human DGAT dimer with seven inhibitors towards the substrate binding pocket. **(G)** Human NPC1L1 structure, with FDA-proved inhibitor ezetimibe targeting the cholesterol binding site.

Increased *de novo* synthesis of fatty acids is a hallmark of cancer cell expansion (Mashima et al., 2009). Several key enzymes involved in the *de novo* synthesis pathway, such as ACLY, ACC, and FASN, are significantly up-regulated, suggesting that these enzymes may be potential drug targets for inhibiting cancer progression (Figure 2; Table 2).

**TABLE 2:** Potential pharmacological targets and related inhibitors targeting fatty acid synthesis.

Drug target	Notable inhibitors	Inhibitor description	IC50	Development status	Related diseases	Chemical st8ructure	References
SLC25A1 (CIC)	CTPI-2	—	3.5 μM	Preclinical Stage	Steatosis, Obesity	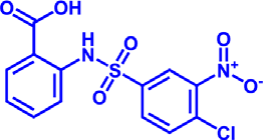	Tan et al. (2020)
SLC25A1 (CIC)	BTA	First-generation inhibitor	—	Preclinical Stage	Solid cancer	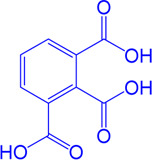	Catalina-Rodriguez et al. (2012)
ACLY	Bempedoic acid	An prodrug; Converted to an active drug in liver	29 uM	FDA approved	Hypercholesterolemia, Mixed dyslipidemia, Statin intolerance	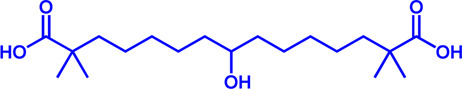	Feng et al. (2020); Masana Marin and Plana Gil, (2021); Ray et al. (2019)
ACLY	Hydroxycitric acid	Natural product from Garcinia	—	Phase 4	Obesity, Diabetes	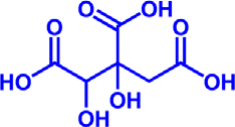	Jena et al. (2002)
ACLY	SB-204990	—	—	Preclinical Stage	Hypolipidaemic	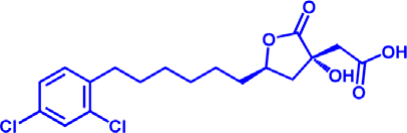	Feng et al. (2020); Pearce et al. (1998)
ACLY	NDI-091143	High-affinity	2.1 nM	Preclinical Stage	Thyroid cancer	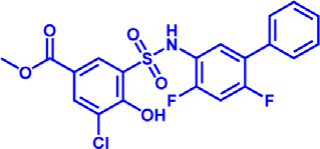	Huang et al. (2022); Wei et al. (2019)
ACC	ND-630 (Firsocostat)	Reversible, highly specific	—	Phase 1	Hepatic Steatosis; Obesity	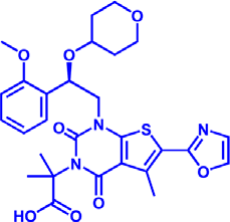	Alkhouri et al. (2020)
ACC	TOFA	Allosteric inhibitor	—	Preclinical stage	Ovarian cancer; Prostate cancer	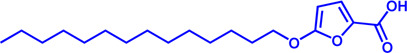	Guseva et al. (2011); Li et al. (2013); Wang et al. (2009)
ACC	PF-05221304	Liver-specific	—	Phase 2	NASH	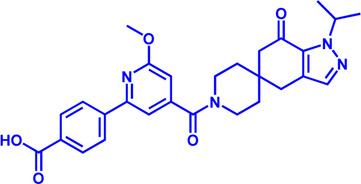	Ross et al. (2020)
ACC1	soraphen-A	—	—	Preclinical stage	Prostate cancer; High-Fat Diet-induced Insulin Resistance, Hepatic Steatosis	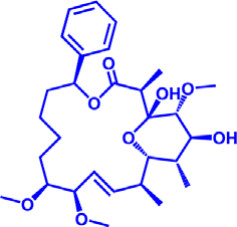	Beckers et al. (2007)
FASN	Cerulenin	Natural inhibitor from Cephalosporium caeruleus	—	Preclinical Stage	Hepatic Steatosis; Solid cancer	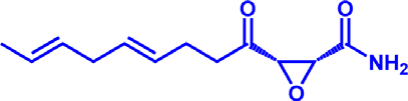	Currie et al. (2013); Menendez and Lupu, (2007)
FASN	C75	Synthetic analog of cerulenin	35 μM	Preclinical Stage	Prostate cancer	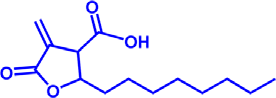	Shimokawa et al. (2002); Thupari et al. (2002)
FASN	EGCG	An phenolic antioxidant from plants such as green tea	—	Phase 2	A wild range of cancers	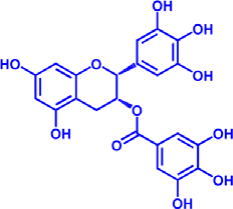	Humbert et al. (2021); Khan et al. (2006)
FASN	Orlistat	The saturated derivative of lipstatin	—	Phase 3	Obesity	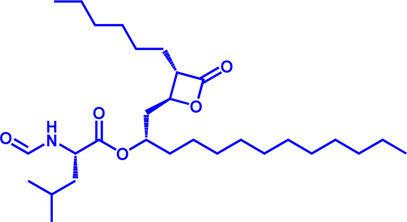	Pemble et al. (2007)
FASN	TVB-2640 Denifanstat	Reversible	0.052 μM	Phase 3	NAFLD; Solid Malignant Tumors	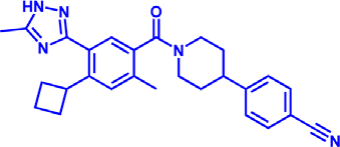	Loomba et al. (2021)
FASN	IPI-9119	Selective and Irreversible	0.3 nM	Preclinical Stage	Castration-resistant prostate cancer	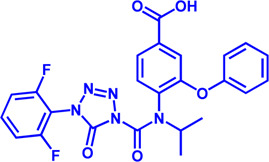	Zadra et al. (2019)
FASN	TVB3664	Reversible	18 nM	Preclinical Stage	Colorectal cancer	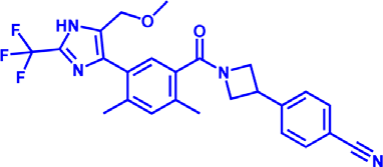	Wang et al. (2022a)
SCD1	A939572	Synthetic Inhibitor	37 nM	Preclinical Stage	Renal cell carcinoma	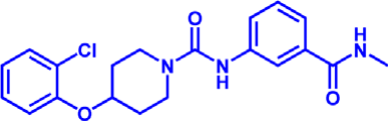	Leung and Kim, (2013); von Roemeling et al. (2013)
SCD1	MK-8245	Liver-selective inhibitor	1 nM	Phase1 clinical trials (NCT00790556) for T2D	Diabetes and Dyslipidemia	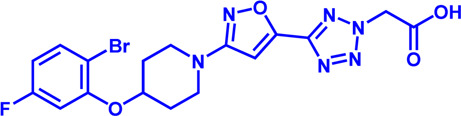	Oballa et al. (2011)
SCD1	CAY10566	—	26 nM	Preclinical Stage	Breast cancer, Lung cancer, Colorectal cancer	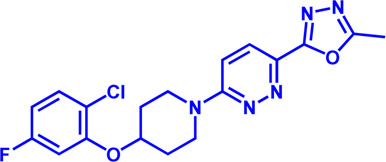	Liu et al. (2007)
DGAT1	AZD-7687	Selective	80 nM	Phase1	Type 2 Diabetes, Obesity	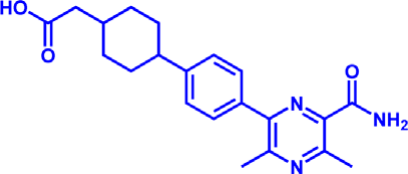	Morentin Gutierrez et al. (2019)
DGAT1	AZD3988	—	6 nM	Preclinical stage	Type 2 Diabetes, Obesity	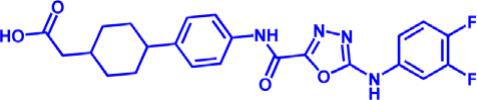	McCoull et al. (2012)
DGAT1	PF-04620110	selective	19 nM	Phase1	Type 2 Diabetes, Obesity	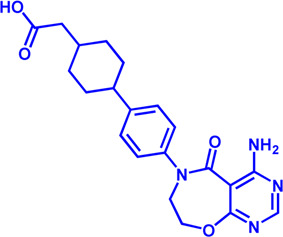	Dow et al. (2011), Lee et al. (2013)
DGAT1	A922500	selective	9 nM	Preclinical stage	hyperlipidemia	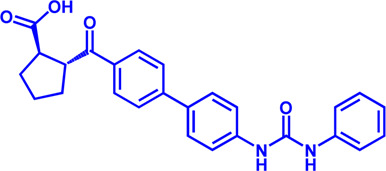	Cheng et al. (2020), King et al. (2010)
DGAT2	PF-06424439	selective	14 nM	Preclinical stage	hyperlipidemia	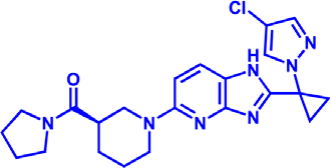	Futatsugi et al. (2015)
DGAT2	PF-06865571 (Ervogastat)	well-tolerated	—	Phase1	NASH, NAFLD	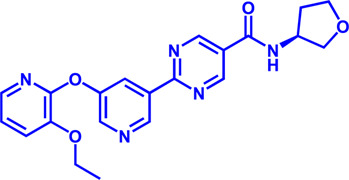	Calle et al. (2021)
DGAT2	JNJ DGAT2-A	selective	—	Preclinical stage	Type 2 Diabetes, Solid cancer	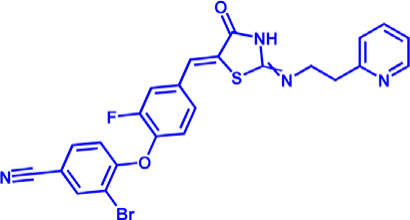	Irshad et al. (2017)

### SLC25A1

SLC25A1 is a channel transporter responsible for shuttling citrate from mitochondrial matrix to cytosol. SLC25A1 is closely linked to various diseases such as myasthenic syndrome (Balaraju et al., 2020). CTPI-2 is the third-generation SLC25A1 inhibitor following the first-generation inhibitor benzene-tricarboxylate and the second-generation inhibitor CTPI-1. Results from *in vitro* studies have shown that CTPI-2 significantly reduces obesity caused by a high-fat diet (Tan et al., 2020) and exhibits antitumor activity (Fernandez et al., 2018), suggesting that CTPI-2 is a novel SLC25A1 inhibitor that is expected to move towards clinical research so far (Figure 2E; Table 2).

### ACLY

ACLY is a key enzyme linking glucose metabolism and lipid metabolism. After citrate is transported from the mitochondrial matrix into the cytoplasm by SLC25A1, it is catalytically cleaved by ACLY to generate acetyl-CoA, which is the substrate for *de novo* synthesis of fatty acids and cholesterol (Feng et al., 2020). Overexpression of ACLY has been reported to be closely related to metabolic diseases such as atherosclerosis, hyperlipidemia (Feng et al., 2020), and cancer (Granchi, 2022). Given the important physiological functions of ACLY, the research on small molecule inhibitors of ACLY has gradually become a hot topic in recent years, especially since the tetrameric structure of ACLY was solved (Wei et al., 2019). ACLY is structurally composed of the Citryl-CoA synthetase (CCS) domain and Citryl-CoA lyase (CCL) domain. Although ACLY inhibitors have been studied for years, few can be used clinically, mainly due to their poor biochemical properties or weak binding. The inhibitors reported so far mainly bind to the CoA binding site and citrate binding site of the CCS domain (Figure 2A; Table 2) (Batchuluun et al., 2022). The former is represented by the inhibitor Bempedoic acid, which was approved by the FDA in 2020 to reduce cholesterol levels in statin-resistant patients and is the only ACLY inhibitor approved by FDA so far (Markham, 2020). The second class of ACLY inhibitors mainly bind to the citrate binding site, represented by Hydroxycitric acid, SB-204990, NDI-091143, and MEDICA 16. Hydroxycitric acid, the first ACLY inhibitor discovered from a natural product, binds competitively to the citrate binding site to inhibit ACLY activity (Jena et al., 2002). However, off-target effects and poor biochemical properties limit its clinical study. SB 204990 (prodrug of SB-201076) is a potent and specific inhibitor of ATP citrate lyase (ACLY). (Pearce et al., 1998). SB-204990 has shown promising efficacy in animal model experiments for the treatment of dyslipidemia such as atherosclerosis (Pearce et al., 1998), but no clinical study data have been performed to date, possibly due to the poor tissue-specific distribution of SB-204990 in humans (Feng et al., 2020). NDI-091143 is a newly identified small molecule inhibitor of ACLY with strong binding properties. It binds next to the citrate binding site of ACLY and prevents citrate from binding to ACLY through allosteric regulation, thereby inhibiting the activity of ACLY (Wei et al., 2019). NDI-091143 represents a new type of inhibitor that is different from previously reported, and has great potential for drug development, although no relevant clinical research data have been reported.

### ACC

ACC is essentially the first enzyme involved in lipid synthesis, containing two isoforms (ACC1 and ACC2) (Wakil and Abu-Elheiga, 2009). ACC1 is located in the cytoplasm and mainly catalyzes the formation of malonyl-CoA from acetyl-CoA for subsequent lipid synthesis (Wakil and Abu-Elheiga, 2009). ACC2 is located in the mitochondrial outer membrane, and also can catalyze the production of malonyl-CoA, but it is functionally biased to negatively regulate FAO (Wang Y. et al., 2022). The reason may be that ACC2 is closer to CPT1. Malonyl-CoA produced by ACC2 is a reversible inhibitor of CPT1 and negatively regulates FAO, as previously described (Wang Y. et al., 2022). ACC is structurally composed of the N-terminal biotin carboxylase (BC) domain, middle biotin-containing carboxyl carrier protein (BCCP) domain, and C-terminal carboxyl transferase (CT) domain (Hunkeler et al., 2018).

Small molecule inhibitors developed for ACC include ND-630, a representative inhibitor that binds to the N-terminal BC domain, and PF-05221304, another representative inhibitor that binds to the C-terminal CT domain (Figure 2B; Table 2) (Alkhouri et al., 2020; Calle et al., 2021). ND-630 (also named Firsocostat) is an ACC inhibitor developed by Nimbus Therapeutics in the United States for NASH and is currently in Phase II clinical research (Alkhouri et al., 2020). The mechanism is similar to AMP-activated protein kinase (AMPK) phosphorylation, which disrupts the dimer formation of ACC subunits, while monomeric ACC cannot catalyze the conversion of acetyl-CoA to malonyl-CoA. PF-05221304 is a liver-preferred ACC inhibitor developed by Pfizer in the United States and has completed a phase II clinical study (Ross et al., 2020). Inhibitor MK-4074 developed by Merck in NAFLD has completed the clinical phase I study (Goedeke et al., 2019). But, no further clinical research has been carried out due to possible side effects of inducing hypertriglyceridemia.

In addition to the above representative inhibitors that have entered clinical research, ACC has some well-studied inhibitors in preclinical research, such as TOFA and Soraphen A. The limitations of TOFA are poor bioavailability and selectivity between ACC and ACLY. Soraphen A, a natural product from soil myxobacterium Sorangium cellulosum, has a similar mechanism of action to ND-630, showing a strong inhibitory effect on eukaryotic (especially fungal) ACC1 (Beckers et al., 2007). Unfortunately, Soraphen A was found to be teratogenic in subsequent studies. ACC, especially the ACC1 isoform, plays an important role in cancer. Targeted inhibition of ACC can exhibit anticancer effects, suggesting that fatty acid synthesis is indispensable for cancer proliferation and metastasis (Crunkhorn, 2016). Although studies have shown that ACC inhibitors (such as CP-640186 of Pfizer Company, Monocyclic derivate-1q of Takeda Company) have initially shown a good inhibitory effect on cancer, no inhibitors have entered the clinical research stage.

ACC inhibitors were shown to be effective in clinical studies, but unexpectedly elevated plasma triglycerides pose a cardiovascular safety risk (Goedeke et al., 2018). Merck Sharp & Dohme’s ACC inhibitor MK-4074 achieved liver targeting but was still discontinued early, likely due to the discovery of elevated triglycerides, as described above. Malonyl-CoA is an intermediate necessary for the synthesis of polyunsaturated fatty acids (PUFA) (Santin and Moncalian, 2018). Inhibition of ACC reduces Malonyl-CoA levels and affects PUFA synthesis, which in turn leads to increased expression of the sterol response element-binding protein-1 (SREBP1) gene and subsequently stimulates very-low-density lipoprotein (VLDL) secretion and elevated plasma triglyceride concentrations (Hannah et al., 2001; Kim et al., 2017).

When using antisense oligonucleotides to inhibit the expression of ACC1 and ACC2 in a rat model of NAFLD, inhibition of ACC1 reduced lipid synthesis and inhibition of ACC2 increased mitochondrial FAO, resulting in reduced hepatic steatosis (Savage et al., 2006). This may provide an attractive treatment for NAFLD/NASH method.

Besides, sequence identity of two isoforms of ACC reaches 75%, but they play different roles in physiological functions (Kim et al., 1998). Thus, combination with lipid-lowering drugs may be the focus of subsequent clinical exploration of ACC inhibitors. One concern is that none of the inhibitors reported so far in the preclinical or clinical stage has reported selectivity for the two isoforms, and may have some side effects. This also implies that future research on ACC inhibitors may require more attention to efficacy and selectivity.

### FASN

FASN catalyzes the endogenous *de novo* synthesis of fatty acids from acetyl-CoA and malonyl-CoA (Lupu and Menendez, 2006). Antitumor effects can be observed when its protein expression is reduced or activity is inhibited by pharmaceutical intervention (Humbert et al., 2021). Therefore, in recent years, FASN has become a much-conceived drug target for cancer therapy (Figure 2C; Table 2). The earliest discovered FASN inhibitors include orlistat (Pemble et al., 2007; Chu et al., 2021), natural product EGCG, cerulenin (Mullen and Yet, 2015), and their synthetic derivatives such as C75, which are unsuitable for clinical use due to their toxicity or poor bioavailability. Several novel small-molecule FASN inhibitors have been developed that inhibit the thioesterase domain. Orlistat inhibits FASN by irreversibly binding to the thioesterase domain (Fako et al., 2014). FASN inhibitors targeting the β-ketoreductase domain have also been developed, and some have recently entered clinical trials, including BI-99179 from Boehringer Ingelheim, and TVB-2640 (also named Denifanstat) from Sagimet Biosciences. TVB-2640 is the most well-studied drug candidate currently in clinical research. Earlier studies have shown that TVB-2640 reduces *de novo* fat acid synthesis by 90% in obese and insulin-resistant individuals. Phase II clinical studies are currently underway in patients with NASH. TVB-2640 is also an FASN inhibitor with significant efficacy on colon cancer, lung carcinoma, breast cancer and glioblastoma treatment (Syed-Abdul et al., 2020; Falchook et al., 2021; Loomba et al., 2021; Batchuluun et al., 2022).

The main side effects of FASN inhibitors are anorexia and weight loss due to accumulation of the lipid metabolism intermediate malonyl-CoA (Turrado et al., 2012). Weight loss theoretically facilitates NASH control, but this effect may be a central nervous system (CNS)-mediated response, which is of great concern (Turrado et al., 2012).

### SCD1

SCD1 catalyzes the production of monounsaturated fatty acids (Lien et al., 2021). A recent study from the Massachusetts Institute of Technology found that reducing SCD enzyme activity in tumor cells or adopting a low-fat diet (especially unsaturated fatty acids) can affect tumor growth (Lien et al., 2021). This makes the development of SCD inhibitors in combination with low-fat-diet a potential cancer treatment strategy. Currently, no SCD1 inhibitor has been approved (Figure 2D; Table 2).

### DGAT

DGAT (two isoforms, DGAT1, and DGAT2) catalyzes the last rate-limiting step in triglyceride synthesis, converting diglycerides and acyl-CoA to triglycerides (Wilfling et al., 2013). Reducing the expression of DGAT or inhibiting activity can effectively reduce diet-induced obesity (Subauste and Burant, 2003). Therefore, the development of DGAT inhibitors has become a research hotspot in terms of obesity. In particular, structure of DGAT has recently been analyzed (Wang et al., 2020), which lays the foundation for the development of structure-based inhibitors. Currently reported DGAT inhibitors include natural products represented by Xanthohumol, AZD-7687, PF-04620110, PF-04620110, A922500, PF-06424439, and PF-06865571 (Figure 2F; Table 2). Although there are currently no FDA approved drugs against DGAT, it is promising to develop drugs for obesity based on DGAT inhibitors. Besides, DGAT1 was first reported in 2020 as a novel target for glioblastoma (Cheng et al., 2020). Glioblastoma is the most malignant tumor in central nervous system, but there are few clinical treatment strategies (Cheng et al., 2020). DGAT1 protects glioblastoma cells from damage caused by excessive FAO by converting excess fatty acids into triglycerides. A922500 is a highly specific, and orally bioavailable inhibitor of DGAT1, with no effect on other acyltransferases. Inhibition of DGAT1 by A922500 effectively prevents the conversion of fatty acids to triglycerides and ultimately inhibits glioblastoma (King et al., 2010; Cheng et al., 2020). However, in-depth clinical data are still needed to determine whether A922500 and other novel DGAT inhibitors have the potential to be therapeutic options for glioblastoma.

### Cholesterol synthesis

Acetyl-CoA is also a substrate for cholesterol synthesis, which can be further processed into hormones, bile acids, and vitamin D (Cerqueira et al., 2016).

### HMGCR

**FIGURE 3:**
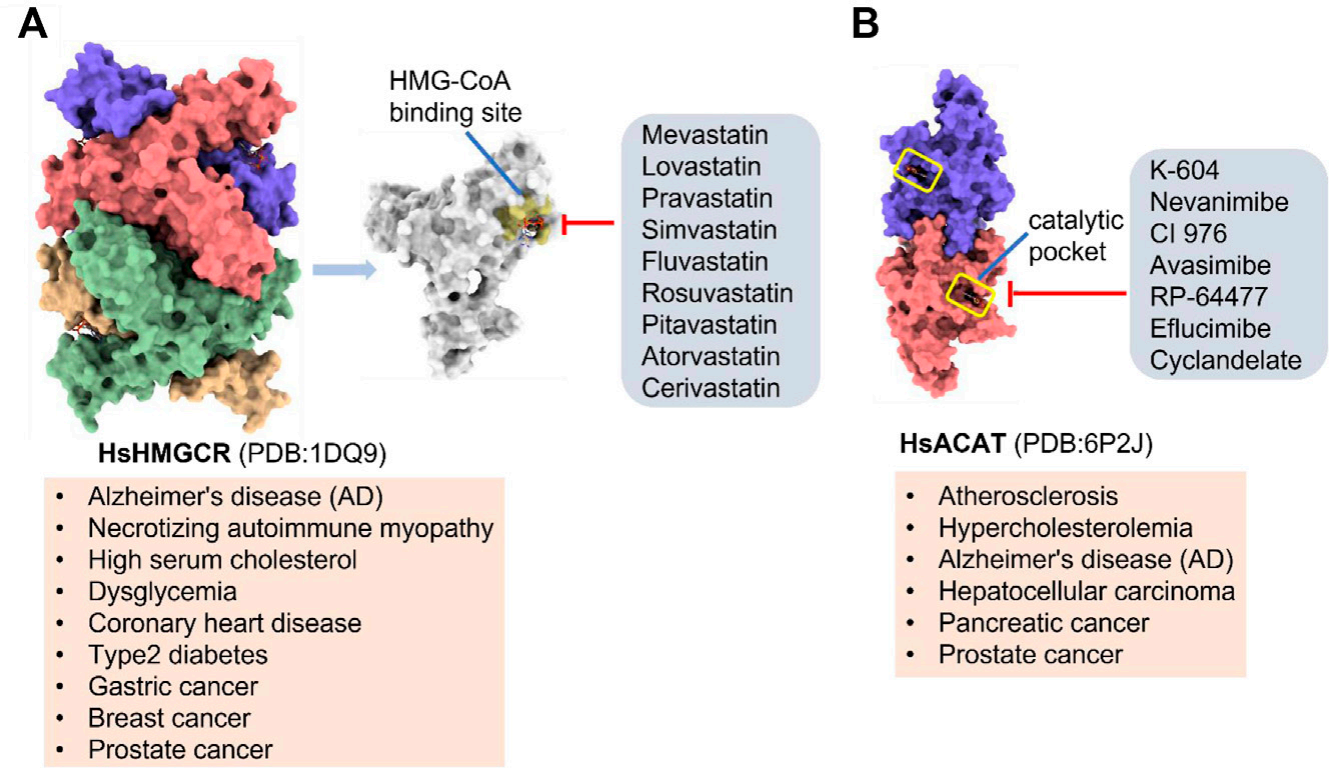
Potential pharmacological targets and related inhibitors targeting cholesterol synthesis and storage, with related diseases are listed under the each structure model. **(A)** Homotetramer structure of human 3-hydroxy-3-methylglutaryl-coenzyme A reductase (HMGCR) and single sununit structure highlighted with the HMG-CoA binding pocket. Statin inhibitors of HMGCR competitively occupy the HMG-CoA binding site and block HMG-CoA from contacting the catalytic center. Several statin inhibitors that have been intensively studied mainly include Mevastatin, Lovastatin, Pravastatin, Simvastatin, Fluvastatin, Rosuvastatin, Pitavastatin, Atorvastatin, Cerivastatin. **(B)** Dimer structure of human Acyl-coenzyme A:cholesterol acyltransferase 1 (ACAT) with catalytic pocket highlighted. Notble inhibitors for ACAT include K-604, Nevanimibe, CI 976, Avasimibe, RP-64477, Eflucimibe and Cyclandelate.

(HMGCR) is the rate-limiting enzyme in cholesterol biosynthesis, which converts HMG-CoA to mevalonate (Istvan and Deisenhofer, 2001). HMGCR is upregulated in gastric cancer (Chushi et al., 2016), glioblastoma (Qiu et al., 2016), and prostate cancer (Ashida et al., 2017). Overexpression of HMGCR promotes the expansion and migration of cancer cells, and knockdown of HMGCR inhibits tumor growth. HMGCR inhibitors have been used for the treatment of drug-resistant solid cancers. Current HMGCR inhibitors in clinical use are mainly statins (Figure 3A; Table 3). Seven FDA-approved drugs include Lovastatin, Pravastatin, Simvastatin, Atorvastatin, Rosuvastatin, Pitavastatin, and Fluvastatin (Omolaoye et al., 2022). The first three inhibitors plus Mevastatin belong to the first generation of statins, derived from fungal products. The latter four plus cerivastatin belong to the second generation of synthetic products. Cerivastatin was later recalled from the market due to a risk of rhabdomyolysis (Furberg and Pitt, 2001). Similar to the substrate HMG-CoA, statins can competitively inhibit HMG-CoA (Istvan and Deisenhofer, 2001). They are relatively safe to take in short term, but long-term use of these drugs can lead to side effects such as rhabdomyolysis and hepatotoxicity (Bjornsson et al., 2012; Karahalil et al., 2017). Therefore, it is also necessary to perform modifications to the drug based on the structure-activity relationship to reduce the side effects.

**TABLE 3:** Potential pharmacological targets and related inhibitors targeting cholesterol synthesis.

Drug target	Notable inhibitors	IC50	Development status	Corresponding diseases	Chemical structure	References
HMGCR	Mevastatin	1 nM	Upgraded to lovastatin	Hyperlipemia; Coronary Heart Disease	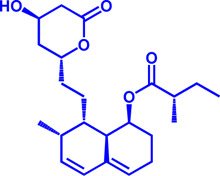	Glynn et al. (2008)
HMGCR	Lovastatin	3.4 nM	FDA approved	Hypercholesterolemia	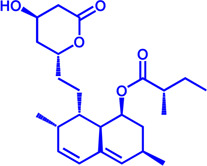	Mulder et al. (2015), Zeller and Uvodich, (1988)
HMGCR	Pravastatin	5.6 μM	FDA approved	Cardiovascular Disease	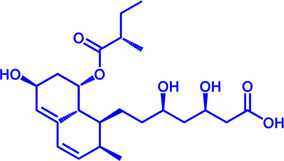	McTavish and Sorkin, (1991)
HMGCR	Simvastatin	95.6 μM	FDA approved	Hypercholesterolemia; Hypertriglyceridemia	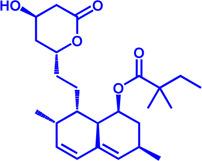	Gryn and Hegele, (2015)
HMGCR	Fluvastatin	8 nM	FDA approved	Hypercholesterolemia	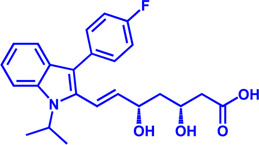	Scripture and Pieper, (2001)
HMGCR	Rosuvastatin	11 nM	FDA approved	Hypertriglyceridemia	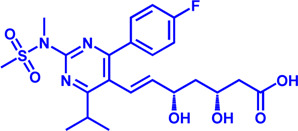	Davidson, (2004), Olsson et al. (2002)
HMGCR	Pitavastatin	5.8 nM	FDA approved	Dyslipidemia; Hypercholesterolemia	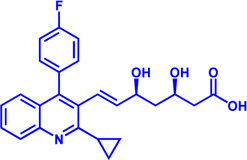	Chan et al. (2019), Hoy, (2017)
HMGCR	Atorvastatin	154 nM	FDA approved	Hypercholesterolemia; Dyslipidemias	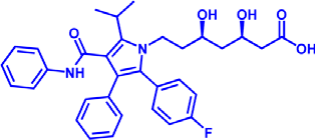	Hu et al. (2021b)
HMGCR	Cerivastatin	6 nM	Withdrawn from the market due to a high risk of rhabdomyolysis	Hypercholesterolemia; Dyslipidemia	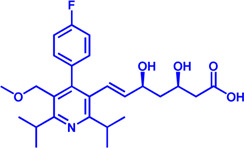	Bischoff et al. (1997), Furberg and Pitt, (2001)
ACAT1	K-604 (selective)	0.45 μM for ACAT1; 102.85 μM for ACAT2	Phase 2 Completed	Atherosclerosis	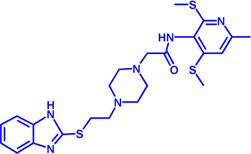	Ikenoya et al. (2007)
ACAT1	Nevanimibe (selective)	52 nM	Discontinued - Phase-II	Adrenocortical Carcinoma; Congenital adrenal Hyperplasia; Cushing syndrome	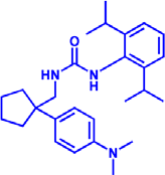	El-Maouche et al. (2020), Long et al. (2020)
ACAT	CI 976	0.073 μM	preclinical	Atherosclerosis; Hyperlipidaemia	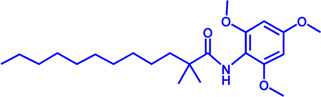	Krause et al. (1993)
ACAT	Avasimibe (CI-1011)	3.3 μM	Discontinued - Phase-III	Atherosclerosis; Hyperlipidaemia	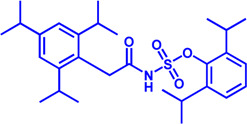	Llaverias et al. (2003), Schmidt et al. (2021), Zhou et al. (2022)
ACAT	RP-64477	503 nM, in human hepatic (HepG2)	phase II	Hyperlipidemia	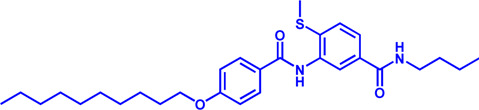	Bello et al. (1996)
ACAT	Eflucimibe	39 nM for ACAT1; 110 nM for ACAT2	Phase-II discontinued	Atherosclerosis; Hyperlipidemia	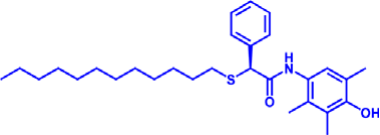	Lopez-Farre et al. (2008)
ACAT	Cyclandelate	80 μM (Rat hepatic ACAT)	Not approved in U.S. or Canada; Approved in Europe	Arteriosclerosis	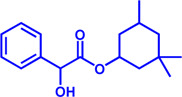	Heffron et al. (1990)

### ACAT

Besides DGAT, ACAT also belongs to the membrane-bound O-acyltransferase (MBOAT) family (Long et al., 2020). ACAT can catalyze excess cholesterol and long-chain fatty acids in cells to synthesize cholesterol esters and store in lipid droplets. ACAT plays an important role in the occurrence and development of diseases such as atherosclerosis (Ohshiro et al., 2011), Alzheimer (Hartmann et al., 2007), and cancer (Yang et al., 2016; Jiang et al., 2019) by regulating cholesterol metabolism. Inhibition of ACAT is expected to be a therapeutic approach to atherosclerosis. In addition, inhibiting ACAT shows a good inhibitory effect on tumor growth (Goudarzi, 2019). Some ACAT inhibitors have entered clinical studies and the results are worth looking forward to (Figure 3B; Table 3).

## Drug targets in lipid oxidation

FAO plays an important role in human metabolism by decomposing fatty acids for energy supply. Long-chain acyl-CoA must pass through the mitochondrial membrane to enter the mitochondrial matrix through the carnitine-palmitoyl shuttle system, which includes CPT1, carnitine palmitoyl transferase 2 (CPT2), and carnitine-acylcarnitine translocase (CACT) (Virmani et al., 2015).

### Acyl-CoA synthase (ACSL)

Free FAs is activated by ACSL to form Acyl-CoA. Cardiac contraction depends on the oxidation of long chain fatty acids to meet energy needs (Carley et al., 2014). Dysfunction of ACSL1 can lead to the accumulation of toxic lipids that endanger heart function. Overexpression of ACSL1 restores normal activation and oxidation of LCFAs and may be a potential option for the treatment of heart failure (Goldenberg et al., 2019). ACSL converts long-chain FAs to fatty acyl-CoAs, which play a key role in FAO, triglyceride, phospholipid, and cholesterol ester synthesis. Triacsin C is a natural inhibitor of ACSL family proteins (ACSL1, ACSL3, ACSL4, ACSL5) from Streptomyces aureus (Kim et al., 2012). Structural of Triacsin C and ACSL complex and in-depth clinical research are needed for the development of Triacsin C-based drugs for ACSL1 related lipid disorders (Table 4).

**TABLE 4:** Potential pharmacological targets and inhibitors targeting FAO.

Drug target	Notable inhibitors	Inhibitor description	IC50	Development status	Related diseases	Chemical structure	References
ACSLl	Triacsin C	An natural inhibitor, from Streptomyces aureofaciens	6.3 uM	Preclinical stage	Lung cancer; Colon cancer; Stomach cancer; Brain cancer; Breast cancer	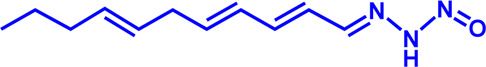	Mashima et al. (2005)
CPT1	Etomoxir	Irreversible; Malonyl-CoA mimetic	5–20 nM (rat liver)	Phase II clinical trial stopped due to hepatoxicity	Leukemia; Glioblastoma	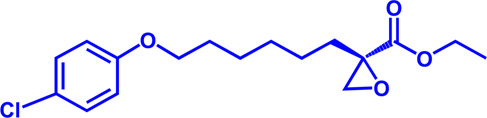	Bristow, (2000); Divakaruni et al. (2018); Lopaschuk et al. (1988); O'Connor et al. (2018)
CPT1, CPT2	Perhexiline (Pexsig)	Inhibit CPT1; to a lesser extent, CPT2	77 μM (rat heart CPT1); 148 μM (CPT1A)	Used primarily in Australia and New Zealand Adverse effects: nausea, hypoglycemia, neuropathy, and hepatitis	Severe angina pectoris	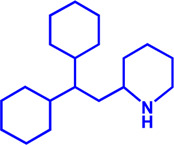	Ashrafian et al. (2007); Ren et al. (2020)
CPT1	ST1326 (Teglicar)	Amino-Carnitine derivative; highly selective for CPT1A; Reversible	0.68 μM (CPT1A)	Discontinued - Phase-II for Type-2 diabetes	Diabetes; Neurodegenerative diseases including Huntington’s disease	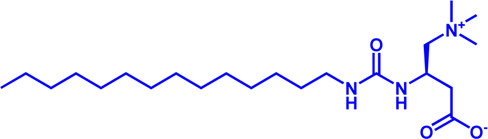	Bertapelle et al. (2022); Conti et al. (2011)
CPT1	2-tetradecylglycidate (TDGA)	Glycidic acid analog; An oxirane carboxylate inhibitor	—	Preclinical Stage, (induce myocardial hypertrophy)	Diabetes	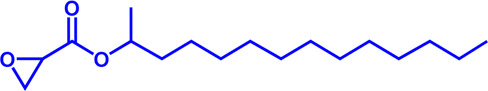	Obici et al. (2003); Schlaepfer and Joshi, (2020); Wolkowicz et al. (1999)
CACT	EN936 (SLC25A20-IN-21)	—	—	Preclinical Stage	—	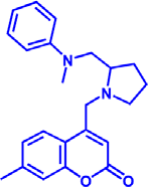	Parker et al. (2017a); Parker et al. (2017b)
VLCAD	Avocadyne	—	—	Phase 1	Acute Myeloid Leukemia; Hyperglycemia	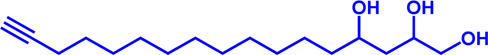	Tcheng et al. (2021a); Tcheng et al. (2022); Tcheng et al. (2021b)
TFPβ	Ranolazine	—	—	FDA approved (NDA #021526)	Chronic Angina	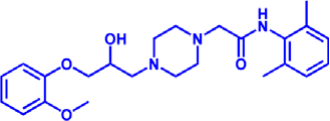	Samudio et al. (2010); Sekine et al. (2022)
TFP	Trimetazidine	—	75 nM	Phase 2 (NCT03273387)	Precapillary pulmonary hypertension; Muscle wasting (cachexia)	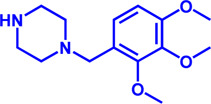	Gatta et al. (2017)

### CPT1

**FIGURE 4:**
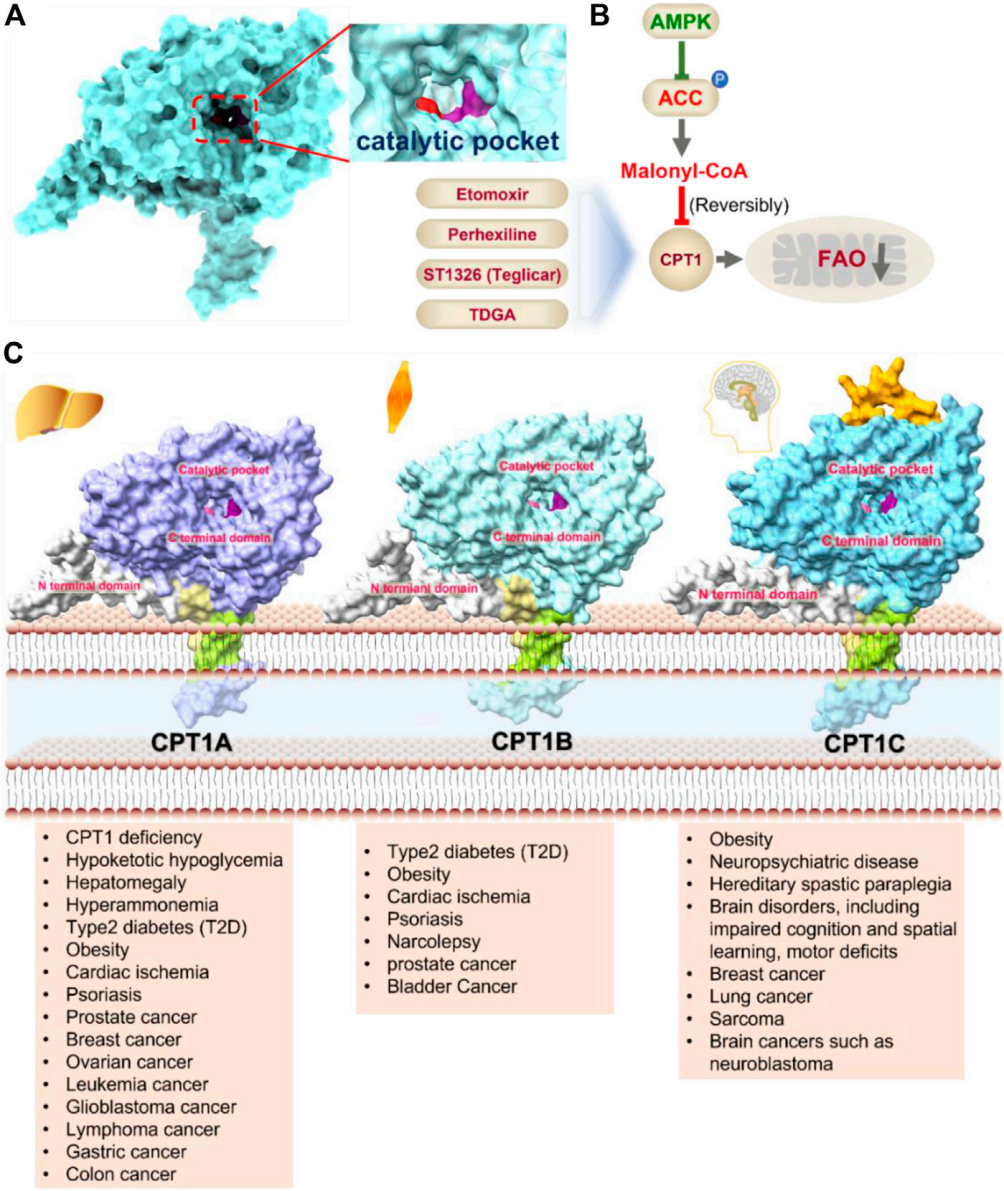
Structure model, notble inhibitors and related disease summary of pharmaceutical target CPT1. **(A)** Structure model of human CPT1 with catalytic pocket highlighted with a magnified view. **(B)** Four notble inhibitors (Etomoxir, Perhexipin, ST1326, TDGA) and physiological inhibitor malonyl-CoA can impair FAO *via* blocking CPT1. Malonyl-CoA level can be regulated by AMPK-ACC axis. **(C)** Structure model for three isoforms of CPT1 (from AlphaFold database), with related diseases are listed under the respective structure model. The liver isoform—CPT1A, the main isoform involved in FAO; the muscle isoform—CPT1B; the brain isoform—CPT1C with little acyltransferase activity. All three isoforms consist of an N-terminal regulatory domain, a C-terminal catalytic domain, and two transmembrane helices, and the catalytic pocket is highlighted by golden color. CPT1C has an extra tail in structure compared to CPT1A and CPT1B, which may be the possible reason why CPT1C plays an important role in neurological diseases.

CPT1 is a key enzyme in fatty acid metabolism, converting fatty acyl-CoA to fatty acylcarnitine (Schlaepfer and Joshi, 2020). The entry of fatty acids into mitochondria is dependent on CPT1, whose activity is regulated by AMPK-ACC axis (Schlaepfer and Joshi, 2020). Malonyl-CoA, catalyzed by acetyl-CoA carboxylase (ACC), is a potent reversible inhibitor of CPT1 (Folmes and Lopaschuk, 2007) (Figure 4A). CPT1 is considered as an ideal drug target for decades. Scientists have been trying to develop activators of CPT1 so that more fatty acids can enter the mitochondria to participate in oxidative metabolism and reduce the accumulation of fat (Dai et al., 2018). For other diseases, small molecule inhibitors of CPT1 may have promising applications. FAO consumes more oxygen than sugar metabolism, so inhibition of FAO can reduce the oxygen demand of cells in specific situations and perform cell protection. Blocking CPT1 has been reported to inhibit the proliferation of a variety of tumors (Wang et al., 2021). It has been shown that inhibition of CPT1 reduces FAO efficiency, which in turn weakens lymphangiogenesis in pathological states such as cancer, while excess lymphangiogenesis favors cancer metastasis (Wong et al., 2017; Li et al., 2021). Carboplatin-based chemotherapy is currently one of the standard regimens for the clinical treatment of cancer, but platinum resistance has been an urgent clinical problem to be addressed for decades with no effective therapeutic strategy established (Brown et al., 2019). High-grade serous ovarian cancer (HGSOC) is the most common and lethal form of ovarian cancer, often diagnosed at an advanced stage, and most patients are platinum-resistant (Cannistra, 2004; Matulonis et al., 2016). A recent study in Cell Reports Medicine showed that targeting cpt1a with platinum-based chemotherapy can improve platinum resistance, although the molecular mechanism is currently unclear (Huang et al., 2021).

Etomoxir is a classical inhibitor of CPT1 that blocks the import of acyl-CoA into the mitochondrial matrix (O'Connor et al., 2018). Phase 1 and Phase 2 clinical trials of Etomoxir were conducted for the treatment of type 2 diabetes and heart failure (Schmidt-Schweda and Holubarsch, 2000). However, etomixir can cause high levels of hepatic transaminases after administration, and can induce severe oxidative stress (O'Connor et al., 2018). The risk of these side effects negates the potential therapeutic benefit of this drug, and Clinical trials have to be stopped due to toxicity and side effects (Table 4).

Perhexiline is an antianginal drug widely used in many countries around the world (except the United States) in the 1970s (Ashrafian et al., 2007). Its principle of action is to target and inhibit the fatty acid transport process (mainly inhibit CPT1, partially inhibit CPT2), and change the energy metabolism substrate of cardiomyocytes from fatty acids to sugars, which can provide more ATP under the same and limited oxygen content conditions (Chong et al., 2016). Although Perhexiline is effective in the initial treatment of angina pectoris, neurotoxicity and hepatotoxicity have been found with long-term treatment (Ren et al., 2020).

Another reversible inhibitor of cpt1a is the amino-carnitine derivative, Teglicar (also known as ST1326), which was invented by sigma-tau and started clinical studies for type 2 diabetes in 2007, and unfortunately ended in 2015 in Phase II clinical trial (Giannessi et al., 2003). Teglicar differs from etomoxir in that it has high selectivity against CPT1A and is reversible, while etomoxir has no significant selectivity against CPT1A and CPT1B and partially inhibits CPT2 (Giannessi et al., 2003). Studies have shown that the combination of ST1326 and ABT199 (Bcl-2 inhibitor) can enhance the anti-acute myeloid leukemia (AML) effect of the latter, indicating that cpt1a may become a potential drug target for the treatment of AML (Ricciardi et al., 2015; Mao et al., 2021). Huntington’s disease (HTT) is a severe neurodegenerative disease, and a Drosophila HTT disease model was used to demonstrate that treatment with the cpt1a inhibitor ST1326 can alleviate the symptoms of HTT (Bertapelle et al., 2022). Although the relationship between mitochondrial energy metabolism and HTT disease is still unclear, the development of safe and effective CPT1 inhibitors may be an effective strategy to slow down the development of HTT disease.

The CPT1 family contains three members: CPT1A (liver isoform), CPT1B (muscle isoform), and CPT1C (brain isoform) (Figure 4B). Sequence identity between human CPT1A and CPT1B reaches 63% overall and 82% near the active site (Ceccarelli et al., 2011). These data suggest that CPT1A and CPT1B may be less selective against small molecule inhibitors. CPT1A has a much higher affinity for carnitine than CPT1B (Ceccarelli et al., 2011). The sequence identity between CPT1C and CPT1A is 55%, but CPT1C has minimal acyltransferase activity (Ceccarelli et al., 2011; Casals et al., 2016). CPT1 plays a crucial role in a variety of diseases, and using CPT1A as a drug target has a very good prospect for drug development (Schlaepfer and Joshi, 2020). Yet, the protein structures of CPT1A and other members of the CPT1 family (CPT1B, CPT1C) are currently unavailable. Therefore, it is urgent to obtain the complex structure with substrate and clarify its catalytic mechanism to provide more effective drugs for the treatment of related diseases. It should be noted that the small molecule development for CPT1 should pay more attention to selectivity as well as efficacy.

### VLCAD

VLCAD catalyzes the first reaction in mitochondrial oxidation of long-chain fatty acids. It is highly expressed in acute myeloid leukemia (AML) patients and is critical for AML cell survival (Tcheng et al., 2021b). Lentiviral knockdown or inhibition of its activity with the specific inhibitor Avocadyne can inhibit the survival and metastasis of AML, but has little effect on the status of normal hematopoietic cells (Tcheng et al., 2021b). Therefore, Avocadyne and its derivatives are likely to show fewer side effects in clinical trials compared to other target inhibitors due to their high selectivity, suggesting that VLCAD is a novel therapeutic target for AML (Tcheng et al., 2021a; Tcheng et al., 2022).

### Mitochondrial trifunctional protein (TFP)

The TFP complex is responsible for the key last three steps in FAO (Liang et al., 2018). Mutations such as HADHA c.1528G > C in TFPα subunit can disrupt the oxidative metabolism of long-chain fatty acids. The excessive accumulation of long-chain fatty acids in mitochondria can lead to diseases such as sudden infant death syndrome (SIDS) (Miklas et al., 2019) and acute fatty liver of pregnancy (AFLP) (Ibdah et al., 1999; Yang et al., 2002; Liu et al., 2017). Clinicopathological analysis showed that HADHA was the most frequently detected in malignant lymphoid tissue, and lowering the expression of TFPα could inhibit the expansion of malignant lymphoma cells, indicating that TFPα was a potential therapeutic target for malignant lymphoma (Yamamoto et al., 2020). TFPβ and TFPα form a complex under physiological state. A recent study confirmed that TFPβ is also highly expressed in malignant lymphoma cells, and treatment with the TFPβ inhibitor ranolazine resulted in a better inhibitory effect than HADHB knockdown (Sekine et al., 2022). Trimetazidine, a potent antianginal drug, inhibits both TFPα and TFPβ, but the specific mechanism still needs to be determined (Kantor et al., 2000; Fould et al., 2010; Hossain et al., 2015; Liang et al., 2018). In conclusion, targeting both TFPα and TFPβ may provide an effective therapeutic strategy for the clinical treatment of malignant lymphoma. More effective inhibitors with higher selectivity need to be developed based on the protein structure and the existing inhibitors for the clinical treatment of malignant lymphoma and lipid disorders.

### ABCD1

FAO can occur both in mitochondria and in peroxisome. Medium and long-chain fatty acids are mainly oxidized in mitochondria, whereas very long-chain FAs (VLCFAs, ≥ C22) are partially metabolized by β-oxidation in peroxisome (Shi et al., 2012). ATP-binding cassette sub-family D member 1 (ABCD1) is a class of ABC transporters located on the peroxisomal membrane, which can transport VLCFAs from the cytoplasm to the peroxisome (Kemp et al., 2001). Dysfunction of ABCD1 leads to metabolic stress caused by the accumulation of VLCFAs in the cytoplasm, leading to X-chromosome-associated adrenoleukodystrophy (X-ALD) (Kemp et al., 2001). Recently, the structure of ABCD1 has been resolved (Le et al., 2022), with its substrate recognition and transport mechanisms revealed, providing a better understanding of the pathogenesis of X-ALD. X-ALD is a rare neurodegenerative disease that primarily affects young children and rapidly leads to progressive, irreversible loss of neurological function and death. In 2021, Skysona received approval for the treatment of patients with early-stage X-ALD carrying the ABCD1 gene mutation and have no HLA-matched hematopoietic stem cell (HSC) donors available (Keam, 2021). Skysona is a one-time gene therapy that uses Lenti-D lentiviral vector transduction *in vitro* to add a functional copy of the ABCD1 gene to the patient’s own HSC cells (Keam, 2021). The effects of Skysona are expected to last a lifetime and do not require the acquisition of a donor HSC from another person. Skysona is the first gene therapy with approval for the treatment of X-ALD (Keam, 2021). The therapeutic goal is to halt the progression of X-ALD to prevent further neurological decline and improve survival in young patients, which is of great significance.

## Conclusion and perspectives

**FIGURE 5:**
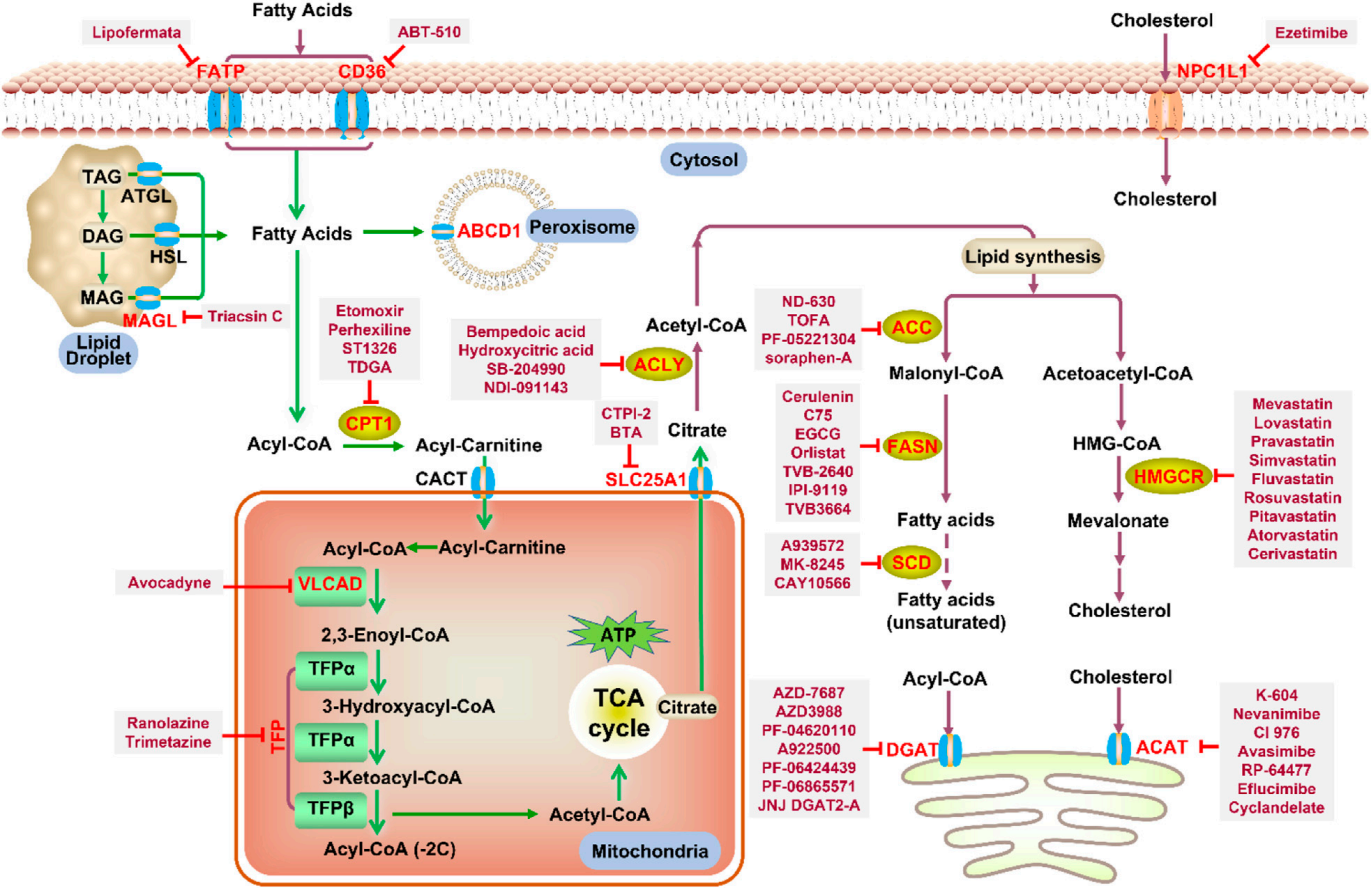
Potential drug targets with related small molecule inhibitors in lipid metabolism. Potential drug targets are highlighted by red color. Abbreviation: TAG, triacylglycerol; DAG, diacylglycerol; MAG, monoacylglycerol.

Disorders in lipid metabolism, which can lead to obesity, hyperlipidemia, atherosclerosis, cancer, and other diseases, seriously threaten the health and have become a research hotspot in recent years. At present, the understanding of the mechanisms of lipid metabolism is still in its infancy. In recent years, a number of anti-tumor drugs targeting lipid metabolism have emerged clinically, and some of them have shown significant anti-tumor effects. The main question now is how to further improve the specificity of these inhibitors without disturbing normal cellular metabolism. For example, CPT1, the most important target of FAO pathway, has different functions and tissue distribution among the three isoforms, and the two isoforms of ACC localize differently in the cell and play different physiological roles. In addition, safety is also an aspect that needs special attention. Although some small molecule inhibitor drugs show outstanding effects, their safety is poor (such as Etomoxir). Therefore, the development of more specific and powerful small molecule inhibitor drugs is a major direction of research in lipid metabolism. This review summarizes research progress on a number of important targets and their inhibitors in the lipid metabolism process (summarized in Figure 5), including marketed drugs, clinical research drugs, and a number of drug candidates in the research stage. It is believed that more and more drugs targeting lipid metabolism will enter the clinic, providing more options for the treatment of lipid metabolism-related diseases and tumors.
